# 

**Published:** 1874-07

**Authors:** 


### CONTENTS.

### COMMUNICATIONS:

Annual Address of the Michigan Dental Association Delivered at Ann Arbor, Oct. 16, 1873 .......... 273
The Brain Power of Man .......... 281
Education .......... 285
Which would be Right .......... 289
Is it good Practice to leave the Dentinal Tubuli open? .......... 293
Principal Cause of Constitutional Derangement in First Dentition .......... 295
Barnum's Coffer-Dam .......... 301
Exposed Nerves .......... 302

### EDITORIAL:

The Rubber Contest .......... 303
Death of Dr. Mendenhall .......... 306
Attention .......... 309
New Instruments .......... 310
American Dental Association .......... 310
Southern " " .......... 311
American Dental Convention .......... 312
Ohio College of Dental Surgery .......... 314
American Academy of Dental Science .......... 314
Kentucky State Dental Society .......... 315
Death from Chloral .......... 316

